# Machine Learning Based Linking of Patient Reported Outcome Measures to WHO International Classification of Functioning, Disability, and Health Activity/Participation Categories

**DOI:** 10.3390/jcm12175609

**Published:** 2023-08-28

**Authors:** Richard Habenicht, Elisabeth Fehrmann, Peter Blohm, Gerold Ebenbichler, Linda Fischer-Grote, Josef Kollmitzer, Patrick Mair, Thomas Kienbacher

**Affiliations:** 1 Karl-Landsteiner-Institute of Outpatient Rehabilitation Research, 1230 Vienna, Austria; rhabenicht@gmx.net (R.H.); peter.blohm@outlook.at (P.B.); gerold.ebenbichler@meduniwien.ac.at (G.E.); li.fischer@me.com (L.F.-G.); kienbacher@rehabzentrum.at (T.K.); 2 Department of Psychology, Karl Landsteiner University of Health Sciences, 3500 Krems, Austria; 3 Department of Physical Medicine, Rehabilitation and Occupational Medicine, Medical University of Vienna, 1090 Vienna, Austria; 4 Department of Biomedical Engineering, TGM College for Higher Vocational Education, 1200 Vienna, Austria; jkollmitzer@tgm.ac.at; 5 Department of Psychology, Harvard University, Cambridge, MA 02138, USA; mair@fas.harvard.edu

**Keywords:** low back pain, international classification of functioning, patient reported outcome measures, random forest, machine learning

## Abstract

Background: In the primary and secondary medical health sector, patient reported outcome measures (PROMs) are widely used to assess a patient’s disease-related functional health state. However, the World Health Organization (WHO), in its recently adopted resolution on “strengthening rehabilitation in all health systems”, encourages that all health sectors, not only the rehabilitation sector, classify a patient’s functioning and health state according to the International Classification of Functioning, Disability and Health (ICF). Aim: This research sought to optimize machine learning (ML) methods that fully and automatically link information collected from PROMs in persons with unspecific chronic low back pain (cLBP) to limitations in activities and restrictions in participation that are listed in the WHO core set categories for LBP. The study also aimed to identify the minimal set of PROMs necessary for linking without compromising performance. Methods: A total of 806 patients with cLBP completed a comprehensive set of validated PROMs and were interviewed by clinical psychologists who assessed patients’ performance in activity limitations and restrictions in participation according to the ICF brief core set for low back pain (LBP). The information collected was then utilized to further develop random forest (RF) methods that classified the presence or absence of a problem within each of the activity participation ICF categories of the ICF core set for LBP. Further analyses identified those PROM items relevant to the linking process and validated the respective linking performance that utilized a minimal subset of items. Results: Compared to a recently developed ML linking method, receiver operating characteristic curve (ROC-AUC) values for the novel RF methods showed overall improved performance, with AUC values ranging from 0.73 for the ICF category d850 to 0.81 for the ICF category d540. Variable importance measurements revealed that minimal subsets of either 24 or 15 important PROM variables (out of 80 items included in full set of PROMs) would show similar linking performance. Conclusions: Findings suggest that our optimized ML based methods more accurately predict the presence or absence of limitations and restrictions listed in ICF core categories for cLBP. In addition, this accurate performance would not suffer if the list of PROM items was reduced to a minimum of 15 out of 80 items assessed.

## 1. Introduction

The World Health Organization (WHO)’s International Classification of Functioning, Disability and Health (ICF) is a standardized reference system and the common basic language for classifying a person’s functioning and health associated with one or several health conditions [1,2]. It is an important framework that enables the comparison of health information from both the patient’s and a healthcare professional’s perspective [3]. The WHO-ICF thereby classifies impairments in structures and functions, limitations in activities and restrictions in participation, considering personal and context factors. Although this classification system has been adopted almost worldwide as the basis of medical rehabilitation, with the selected categories having become part of the International Statistical Classification of Diseases and Related Health Problems ICD-11, the use of the WHO-ICF outside of institutionalized rehabilitation care, i.e., in primary medical and specialist medical care, is still rare. This is despite the fact that most of the diseases managed in the primary and secondary health sector are non-communicable and chronic, and despite the availability of WHO-ICF core sets. WHO-ICF core sets refer to a brief list of those categories that would most likely affect a person’s functioning and health state when diagnosed with a health condition. In primary and specialist medical care, however, the ICF is not generally used. Instead, patient-oriented, fully validated outcome measures, which are assessed using questionnaires, are increasingly used to assess a patient’s disease-related health state and quality of life. The information gained from these questionnaires is more and more frequently considered in the medical decision-making process regarding the best medical management of a disease, particularly in older persons. Many persons diagnosed with one or several chronic health conditions would likely benefit from medical rehabilitation [4]. Endorsed by the WHO’s resolution on strengthening rehabilitation in health systems and to facilitate the selection process of those patients in need of rehabilitative and secondary/tertiary preventive health interventions, it would be highly desirable to have a patient’s health information, particularly health information that covers their activity and participation domain, ready for medical management and communication in a standardized way.

The linking of PROM-rated information to ICF core sets has been suggested by Cieza [1,5], and rules have been established and updated regularly [1,6,7]. Notably, the PROM-collected information refers predominantly to subjectively perceived impairments in functions, limitations in the performance of activities and restrictions in participation; these describe the degree of person’s disability. These manual linking rules are limited in usability because they would have to be applied through an elaborate process involving different raters and a dialogue in case of disagreement. In addition, it may be that not all categories are well covered, leading to scaling problems, and many ICF categories are covered by only one item within these linking rules. If, however, linking was available using automatic algorithms, resources could be saved and the decision-making process considering the need for rehabilitation could be footed on the WHO-ICF classification [1,8,9]. In addition, the results of the linking process can be communicated to a patient in real time following the collection of the PROMS. This provides an opportunity for the patient to give feedback on the accuracy of the linking outcome, which fosters greater patient participation in their treatment process, and can lead to an increase in data quality.

Among the different automatic linking strategies possible, linking rules based on Rasch analysis [1,9,10] focus on the development of quantitative measurements by aggregating ICF categories into higher domains; these would not, however, allow one to classify individual ICF categories separately. The Rasch model proposed by Cieza [1] could be used for classifying separate ICF categories, but it requires predefined linking rules by experts to accurately link PROMs to different ICF categories, thereby limiting its usability in daily medical care. Machine learning (ML) techniques appear particularly appropriate, if information from widely used PROMs is to be fully and automatically linked to the ICF core categories. Our research group recently proposed a linking method for ICF categories of the comprehensive core set for LBP by developing random forests (RF) for each ICF category [8]. Even though the accuracy of the 11 RFs was satisfactory for some of the ICF categories, the algorithms require further improvement to allow for accurate linking to all ICF categories. Therefore, this research sought to further improve the fully automatic linking process using ML methods. As state-of-the-art of ML methods constantly change, different methods were compared to each other and considered for the further development of the linking methods. Specifically, it was of interest whether we would be able to accurately predict the ICF categories within the activity and participation component in the example of the brief core set for LBP. The goal was to achieve a constant and improved performance over all 12 included ICF categories through developing ML methods with a high number of PROMs and compare them with other linking algorithms.

The comprehensive assessment of a person’s functioning and health utilizing a large set of different PROMs may be boring for a patient and therefore negatively affect their willingness to complete PROM questionnaires with an appropriate level of attentiveness, which would consequentially have negative impacts to the data collection quality [11]. Therefore, a further aim of this study was to identify the quantitative and qualitative minimum PROM item set that would best substantiate the performance of the prediction methods in order to achieve higher feasibility. By providing results for different sets of PROMs with higher and lower feasibility, this study presents different solutions for different use cases, either for high accuracy and good feasibility or for even higher feasibility with slightly lower accuracy.

## 2. Material and Methods

### 2.1. Ethics Statement

This study conformed to the ethical principles of the World Medical Association Declaration of Helsinki and was approved by the Ethics committee of the city of Vienna, number “EK_11_181_VK_NZ”. All participants received oral and written information about the study and provided written consent.

### 2.2. Participants and Study Design

Between February 2020 and September 2021, all cLBP patients scheduled to start with an outpatient rehabilitation at the Karl-Landsteiner Institute of Outpatient Rehabilitation Research and who gave consent to the use of their data for study purposes were elected for this study.

A total of 805 patients with chronic non-specific cLBP (494 females = 61%), between 16 and 79 years of age (mean: 48.8) completed an examination performed by a Physical and Rehabilitation Medicine specialist at the start of their therapy process, during which time they were informed about the therapy process and were checked for eligibility. After the medical examination, patients needed to fill out several questionnaires. Patients were asked to attend a psychologist’s examination to assess their mental health status and to classify their functioning, disability, and health with the ICF questionnaire. All the included cLBP patients suffered from LBP but were otherwise healthy.

### 2.3. Measures

#### 2.3.1. Demographics and Pain Level

Participants filled out a demographic checklist that assessed their gender, age, body mass index (BMI), educational level, marital status, employment status, and their ability to work. Another part of the demographic check list was the assessment of their pain level (perceived pain level at examination day, measured on a visual analog scale from 0 (no pain) to 100 (most severe pain imaginable)) [12] and the following questions about the pain history of the patient: pain duration (<1 year, 1–2 years, 2–5 years, 5–10 years, >10 years); start of current pain period (0–6 weeks, 7–12 weeks, >12 weeks); pain present before current pain period (yes/no); and previous stay at health or rehab facility (never, 1 time, 2–3 times, 4–5 times, >5 times).

#### 2.3.2. ICF Core Set for LBP

The ICF comprehensive Core set for LBP was assessed by trained clinical psychologists during a semi-structured psychological interview within the first few weeks of therapy. The ICF five-point scale (0 = no impairment, 1 = mild, 2 = moderate, 3 = severe, 4 = complete) was used to determine the severity of a patient’s problem within a category. Two additional response options were available: “not specified” and “not applicable”. The 12 ICF categories of the activity and participation component listed in the brief core set for LBP were used as target for the linking process in this study. These 12 categories were as follows: d240 “Handling stress and other psychological demands”, d410 “Changing basic body position”, d415 “Maintaining a body position”, d430 “Lifting and carrying objects”, d450 “Walking”, d530 “Toileting”, d540 “Dressing”, d640 “Doing housework”, d760 “Family relationships”, d845 “Acquiring, keeping and terminating a job”, d850 “Remunerative employment”, and d859 “Work and employment, other specified and unspecified”.

#### 2.3.3. Questionnaires

Following a literature review, a team composed of psychologists and physicians discussed the most suitable questionnaires for the use of linking and predicting ICF core sets for LBP with items from other questionnaires. The following questionnaires were chosen and used as PROMs in this study:

Roland–Morris disability questionnaire (RMQ): 24 items that measure pain-related disability resulting from LBP. Every item is a statement that a patient can agree or disagree with. The sum score ranges from 0 to 24, with higher scores indicating higher disability levels. [13,14]. Previous studies and linking rules showed that several RMQ items can be linked to the LBP brief core set of the ICF [15,16].

Pain Disability Index (PDI): seven items that measure disability and impact of pain in seven domains of life on a scale from 0 (no disability) to 10 (total disability). The sum score has a range between 0 and 70, with higher values indicating higher disability levels [17,18].

European Quality of Life 5 Dimensions 5-Level (EQ5D): five items that measure a patient’s health state within five dimensions, with one additional item (EQ5D VAS) recording a patient’s self-rated health on a Visual Analogue Scale (VAS) from 0 (worst health you can imagine) to 100 (best health you can imagine) [19].

Hospital Anxiety and Depression Scale (HADS): fourteen items, consisting of seven questions for assessing anxiety and seven questions for depression. The sum score has a range between 0 and 21, with higher values indicating higher anxiety/depression levels [20].

Avoidance endurance questionnaire (AEQ): seven items from the pain persistence sub-scale were used as well as two additional items [21]. The sum score has a range between 0 and 6, with higher values indicating higher pain persistence behavior.

Subgroups for Targeted Treatment Back Screening Tool (START): nine items (eight items with yes/no answers and one item with a range from 0 to 5) that are used to identify subgroups of patients with LBP based on various functional, psychosocial, and comorbid factors for subgrouping [22,23].

### 2.4. Data Preparation and Selection of Classifier

Descriptive statistics were used to summarize the patients’ characteristics and to describe the patients’ health status based on the scores of the PROMs used for the dataset in this study. The percentage of patients showing limitations/restrictions within each ICF category was visualized using barplots. All statistical analyses were performed in the R environment for statistical computing [24]. Package ggplot2 [25] was used for visualizing the results.

The focus of the linking process was to distinguish the presence or the absence of a category listed in the ICF core set for LBP. Patients’ responses on the ICF five-point scale were dichotomized into 0 = “no impairment” and 1–4 = “impairment”. This dichotomization process is in accordance with published recommendations [8,26,27]. The two additional responses for the assessment of ICF categories (“not specified” and “not applicable”) were treated as missing.

For each ICF category, a separate linking method was calculated with the above-mentioned PROMs as predictors and with one of the ICF categories as response. Each of those 12 methods therefore had 80 PROMs as predictors and one ICF category as response. Prior to training the prediction methods, missing values in predictors were inputted using the k-nearest-neighbor method [28,29]. This step was taken because in some of the tested ML methods the built-in solution to this problem is to skip missing data, which can cause performance degradation and biased outcomes [29]. The “VIM-Visualization and Imputation of Missing Values” package [30] was used for k-nearest-neighbor imputation.

Among the different ML methods that were tested using the whole set of PROMs, the following ones were deemed appropriate: logistic regression, logistic regression with splines on metric variables, logistic lasso, ridge regression, support vector machines, decision trees, RFs, and xgboost. Out of this selection of methods, the RFs revealed the best performance and were therefore used for further analysis.

### 2.5. Development and Tuning of RFs

RF is a supervised ML method that aggregates the results of many randomly constructed decision trees. As with all tree-based methods, this tree building process involves recursively partitioning the dataset into two groups based on a certain criterion until a predetermined stopping condition is met. One typical split criterion is the Gini index, which is used to measure how good a split between two groups is. A lower Gini index indicates a better split and is therefore preferable for a split within a decision tree [31]. For the RF method, each tree is built on a different bootstrap sample with different random sets of variables and random subsets of the training data. Due to this randomization, RFs are robust to overfitting and can achieve high accuracy with relatively small processing times. Other advantages of using RF are that they are able to handle outliers and imbalanced data, and they are a good fit when the data is highly dimensional, which refers to a high number of variables within the dataset. The drawbacks of the aggregation used in RF is that in contrast to individual decision trees, RFs are not easily interpretable [32,33,34].

For the implementation of the RF method, the “randomForest” package [35] was used; this package uses two different parameters for tuning: “ntree” and “mtry”. While the parameter “ntree” sets the number of trees that are randomly generated during the RF method, the parameter “mtry” defines the number of variables randomly sampled as candidates at each split. As the default value for the number of trees was sufficiently large, only the parameter “mtry” was used for tuning during a 10-fold cross validation (CV) process for each RF [28,36]. Ten-fold CV is a technique used to evaluate the performance of a ML model. The data is randomly partitioned into 10 equal subsets, and the model is trained and tested on each of these subsets in turn. This process is repeated 10 times, with each subset being used for testing once, and the results are averaged to provide an estimate of the model’s performance [28].

The area under the receiver operating characteristic curve (ROC-AUC) [37] was used as criteria to determine the optimum “mtry” value during the 10-fold CV tuning process. The caret package was used for this tuning process of the RF methods [38].

### 2.6. Evaluation of RFs

For evaluation purposes, we use the out-of-bag (OOB) sample evaluation. OOB samples are the instances that are not included in the bootstrap samples used to build each tree in the RF. These OOB samples fall out naturally from the underlining RF model and are then used to estimate the performance of the RF model [28].

Several performance metrics were used to evaluate the RF methods:

(1) The ROC-AUC was used to compare performance of the methods. The ROC-AUC is recommended to investigate imbalanced data as this was the case for most of the ICF categories in this dataset. The ROC AUC defines the optimal balance of sensitivity and specificity and can take a value between 0.5 and 1, where a ROC value of 1 would represent a perfect classifier and a value of 0.5 would mean that the classifier is not better than a random guess. Sensitivity is defined as the true positive rate, whereas specificity is referred to as the true negative rate. For the ROC AUC values, the following considerations can be made: 0.7 to 0.8—fair; 0.8 to 0.9—good; 0.9 to 1—excellent [28,39,40].
(2) It is possible, especially with imbalanced data, that the AUC values show good performance even when either the sensitivity or the specificity values are low. Therefore, specificity and sensitivity are also reported as these metrics provide useful information about the model performance.
(3) Precision provides further information as it is the rate of true positives divided by all positive predictions.
(4) As the harmonic mean of precision and sensitivity, the F1 score was also included as a performance metric.
(5) The overall accuracy, defined as the proportion of correctly predicted instances out of the total number of instances, was also provided as additional information. Due to the fact that most ICF categories in the dataset of this study are imbalanced, it should be noted that accuracy as a metric can be misleading [28].
(6) Cohen’s Kappa coefficient of agreement between a problem observed within a category and a predicted problem within a category was used as a further metric. Scores range between −1 and 1, with negative values indicating worse performance than random chance and positive values indicating better performance than random chance [28]. Values exceeding 0.2 suggest fair agreement; those exceeding 0.4, moderate agreement; and those exceeding 0.6, substantial agreement [41,42].

### 2.7. Reduction in PROM Items Based on Variable Importance Measures

A second version of the RF was fitted using recursive feature elimination (RFE). This technique [43] uses the variable importance of the previously built RFs, which were fitted for the whole set of variables, and then removes the least important predictors. RFE removes variables from an RF until the optimal subset of predictors is obtained. This optimal subset was then used for the second version of the RF, and figures were created to illustrate change in performance for a different number of predictors. The “Rfe” function of the “caret” package [38] was used for the implementation of RF based on RFE.

The RFE method is compatible with RFs because RF use the full set of predictors for the initial method, while these RFE tend to not exclude variables from the prediction equation. Therefore, RFE can be used to conduct a post hoc pruning of variables that are irrelevant or not essential for the performance of the RF. Another reason for the compatibility of RF with RFE is that RFs have good internal methods for measuring variable importance [28].

### 2.8. Data Availability

The anonymized datasets generated and analyzed during the current study are available from the corresponding author upon reasonable request.

## 3. Results

Data from a total of 805 persons diagnosed with chronic LBP were collected to train the RFs. Of these, a complete dataset of values was available from 508 patients, only one variable was missing from 133 patients, and more than one variable was missing from 164 patients, with item PDI5 being absent most often (88 times) due to the intimacy of the question. The RFs used 80 predictors for training the RF methods in addition to the 12 ICF categories. These 80 predictors included 24 RMQ items, seven PDI items, nine AEQ items (seven PPS items and two additional items), nine START items, six EQ5D items (five categories and VAS), 14 HADS items and 11 demographics or other variables (age, gender, and pain level VAS), education, marital status, employment status, ability to work, pain duration, the start of current pain period, pain present before current pain period, and previous stay at health or rehab facility. The demographics and baseline characteristics of all included patients are presented in Table 1.

Back pain intensity as rated by the 805 (61% females) patients on VAS revealed moderate levels (mean: 42.61; standard error (SE): 0.91). Overall, their pain related health was impaired in a mild way as derived from low RMQ (mean: 5.63; SE: 0.17), PDI (mean: 22.00; SE: 0.56), high EQ5D (mean: 0.76; SE: 0.01), and EQ5D VAS scores (mean: 64.75; SE: 0.72). The HADS scores indicted mild depression and anxiety levels (mean depression: 4.93; anxiety: 6.34). Furthermore, AEQ assessments revealed moderate pain persistence behavior (mean AEQ PPS: 3.35; SE: 0.04), and 66 percent of all patients were considered “medium risk” for pain chronification by the START back screening tool (low: 14%; high: 11%). Most of the participants where employed (67%), married (52%), and had professional training as highest education form (40%).

**Figure 1:**
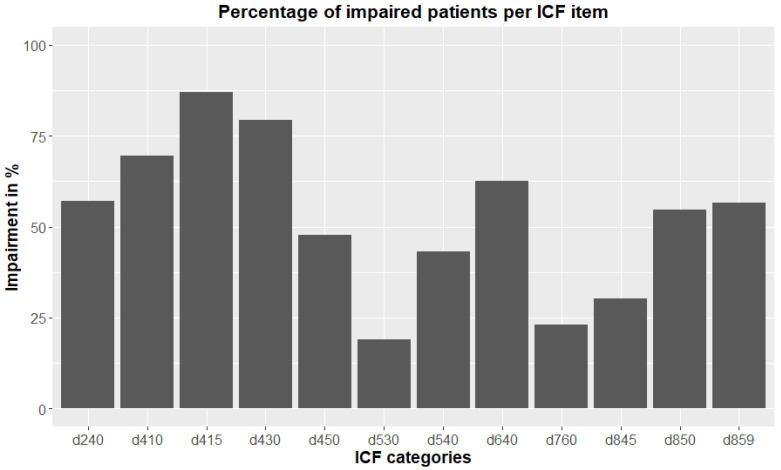
This figure shows the percentage of impaired patients for each ICF category.

The prevalence rates of limitations/restrictions, assessed during psychological interviews, were unbalanced, with high prevalence rates observed for the categories d410, d415, d430, and d640, and low ones for the categories d530, d760, and d845 (see Figure 1). The highest percentage of patients with a limitation/restriction was observed for category d415 “maintaining a body position”, and the lowest one for category d530 “toileting”. The categories d240, d450, d540, d850, and d859 were all fairly balanced, with percentages between 40 and 60 percent.

### 3.1. Performance of the Linking Methods

**Figure 2:**
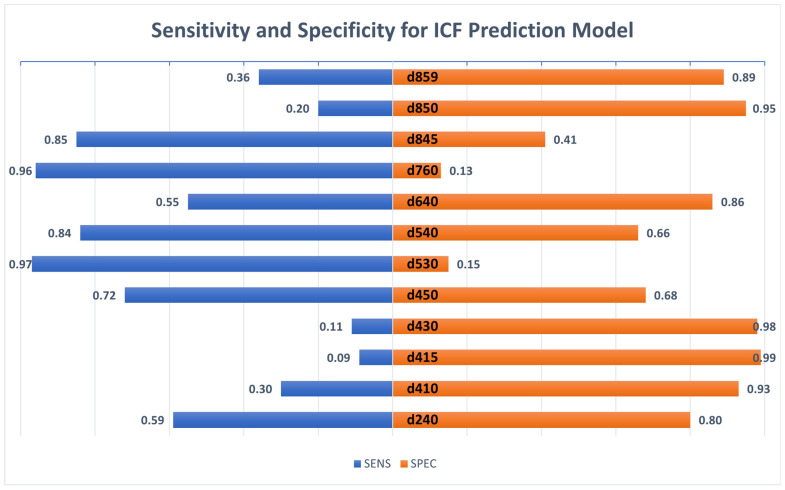
In this figure, sensitivity and specificity are shown in comparison for the RF method of each ICF category.

Results of the RF prediction methods are shown in Table 2. Overall, the RFs and RFE-based RFs were comparable in linking performance and showed better performance than that observed for a recently published one [8] (see Table 3). Both the RFs and the RFE-based RFs revealed an overall fair to good performance for all ICF categories with AUC values ranging between 0.73 and 0.81. The two ICF categories d540 and d640 had the best performance, while the ICF categories d850 and d859 showed the lowest AUC scores. Considering the prevalence rates within the training data and other performance metrics, ICF categories that were found to be highly unbalanced within the training set showed either low sensitivity or low specificity scores and low kappa scores. Noticeably, ICF categories that indicated disability in a high proportion of participants (ICF d410, d415, d430, and d640) revealed low sensitivity scores (d410: 0.30; d415: 0.09; d430: 0.11), whereas ICF categories with a low prevalence of disability revealed low specificity scores (d530: 0.15; d760: 0.13; d845: 0.41). Kappa scores were in a range between moderate and good for most ICF categories, except for categories that showed very low sensitivity or specificity scores (d415, d530, d760, and d850). Precision scores were moderate to good for all ICF categories, with the lowest score for category d430 (0.53), and the highest one for d530 (0.82) (Table 2, Figure 2).

F1 scores found the majority of the predicted ICF categories to be moderate to good. Categories d415, d430, and d850 revealed low F1 scores and low sensitivity scores. The accuracy values of the RFs were good for all ICF categories, with all accuracy scores exceeding 0.70 (Table 2).

An inspection of confusion matrices of each RF, which are shown in Table 4, found those categories, which displayed either low sensitivity or low specificity values, less predicted in an accurate way within the underrepresented class. Although the ICF category d415 classified nearly all cases (778 out of 784) as “not impaired” even though there were 79 actual “impaired” cases in the dataset, other categories with high imbalance showed similar problematic distributions of correct predictions (d430, d530, d760, and d850).

When compared to the traditional RFs, the RFs with RFE revealed minimal improvements in AUC scores for 7 out of the 12 predicted ICF categories. It is worth noting that none of the categories showed a decline in AUC score after RFE. RFE was further associated with minor improvements for other metrics (e.g., sensitivity for d430), but in some cases, this procedure caused minor declines in scores (e.g., F1 score for d415).

### 3.2. Reducing the Number of Items Utilized for the Linking Process

Inspecting those PROM items that were most relevant to the prediction process of the different WHO-ICF core activity/participation variables, as derived from the variable importance measures, suggests that the two items “pain intensity” (measured on a VAS scale) and “patients’ age” were among the 10 most important variables in each of the methods investigated.

**Figure 3:**
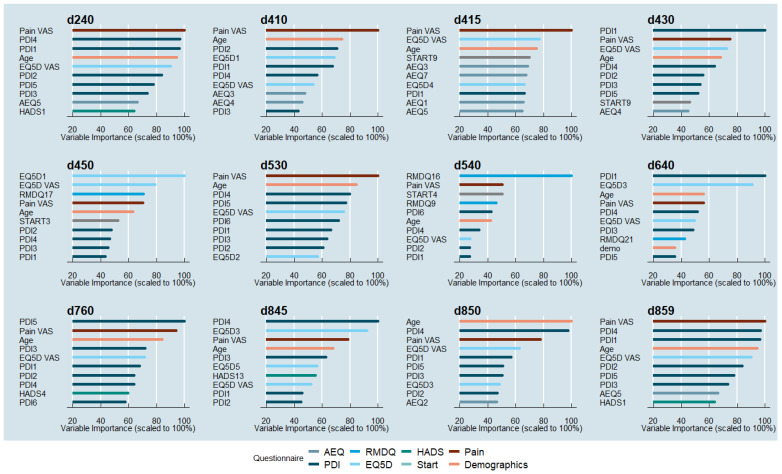
Visual representation of variable importance for the RF method of each ICF category. Variables are colored based on the type of questionnaire.

PROM items of importance utilized by the RF for predicting the WHO-ICF core activity/participation categories were most frequently represented in the PDI. In particular, at least one of the PDI items 1–4 (1 = Family/Home Responsibilities, 2 = Recreation, 3 = Social Activity, 4 = Occupation) were among the 10 most important predictors for all RFs. The EQ5D VAS health index was represented in 11 out of 12 methods investigated. The other five items of the EQ5D questionnaire were important for at most three RF. Items from the AEQ questionnaire were important for 6 out of the 12 ICF categories predicted (d240, d410, d415, d430, d850, and d859), while only a few items (HADS: 1, 4, and 13; START: 4 and 9; and RMDQ: 9, 16, 17, and 21) from the other questionnaires were among the most important variables for any of the RFs. Figure 3 shows the 10 most important variables for the prediction of each ICF category.

**Figure 4:**
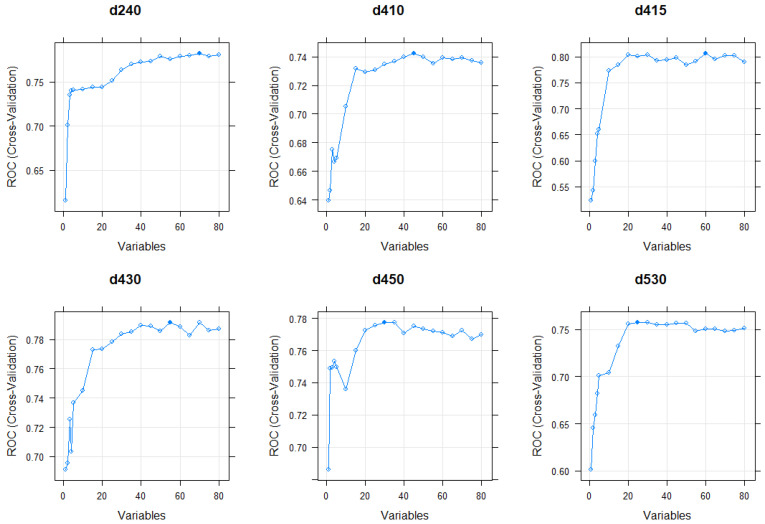
This figure shows the optimal number of variables for the RF methods of ICF categories d240, d410, d415, d430, d450, and d530 based on recursive feature elimination. The optimal number that was selected for the second version of the reported RF methods is marked as a filled blue dot.

**Figure 5:**
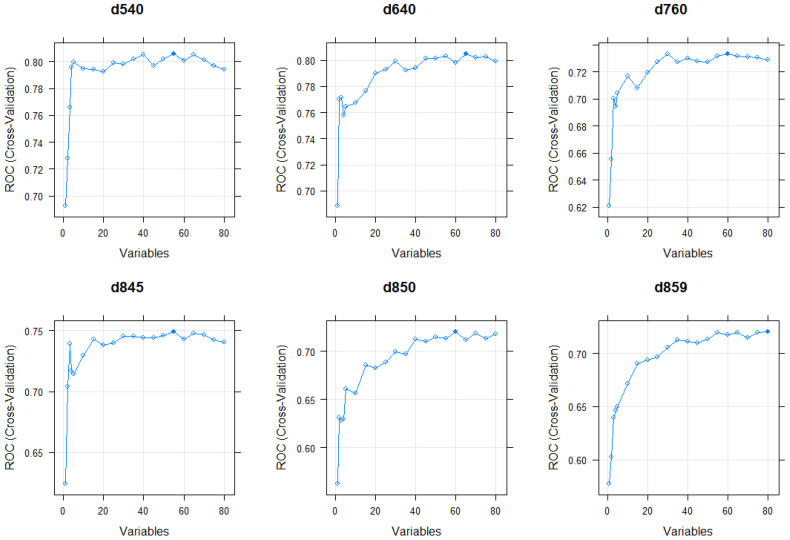
This figure shows the optimal number of variables for the RF methods of ICF categories d540, d640, d760, d845, d850, and d859 based on RFE. The optimal number that was selected for the second version of the reported RF methods is marked as a filled blue dot.

Findings from RFE for the RFs suggests that the number of PROM items necessary to successfully predict each activity/participation LBP WHO ICF core category is optimized at 60 to 80 variables for most of the ICF categories. Nevertheless, when the number of variables was reduced to 20–40 per ICF category, the RFs maintained their predictive validity. Visualizations of the optimal number of variables for each ICF category are shown in Figure 4 and Figure 5.

Subsequent analyses that considered a set of 24 PROM items deemed to be of importance for successful linking revealed a similar performance of the RF compared to the full data PROM item set (80 items) (Table 5). When the two variables “patient age” and “pain intensity” (VAS) were added to the minimum subset of PROMs, the performance of the RF was found to be either similar for the 24-PROM-item set or slightly decreased for the 15-item set for all WHO-ICF core categories. The time required to complete a minimum PROM data set including 24 items would be reduced to ten minutes compared to the 25 min that were needed by patients completing the full PROM data set.

## 4. Discussion

The aim of this study was to further optimize the performance of fully automatic linking processes that allow extracted information from PROMs to accurately predict ICF categories that indicate a limitation or restriction, as well as to find a minimal set of PROMs necessary without compromising performance. Patients with LBP and the WHO-ICF brief core set for LBP were used in this study. The main findings of this study revealed that

- the modified RFs with and without feature extraction achieved fair to good accuracy and a consistently fair to good performance for all the 12 ICF core categories investigated with no major differences between each other. These modified ML linking methods performed better than a previously published one.
- A minimum data set of PROM items (24 items) that allowed for automatic linking to the WHO ICF activity/participation core categories for LBP at a performance that was similar to that of the full PROM data set was identified. Additionally, the automatic linking performance was only slightly decreased when a subset of 15 important PROM items was considered. The time required for patients’ to complete the questionnaires could be considerably reduced from 25 min for the full set to less than 10 min for the set of 24 items.

The performance testing and tuning of our further developed ML methods is dependent on the sample tested and thus has to be representative for the population investigated [44,45]. In fact, this study sample revealed high prevalence rates of limitations and restrictions in several ICF categories (d410, d415, d430, and d640), and moderate rates of limitations in five out of eight of the other ICF categories (d240, d450, d540, d850, and d859). These results are consistent with those published for outpatients with chronic idiopathic LBP, who typically reported moderate pain intensity levels in previous research [8,46,47]. The limited activity and restricted participation ICF profiles are also similar to those published for other chronic conditions [48]. However, specific back pain patient groups, such as those with osteoporosis and acute vertebral fracture, spinal stenosis, or acute disc herniation, may report highly intense pain levels. In such groups, the limitation and restriction profiles may indicate clearly higher disability levels compared to the chronic idiopathic LBP patient group. Therefore, including other LBP patient groups with higher pain and disability levels in the study sample could be considered for further research as it may positively influence the performance of ICF categories that showed low prevalence rates of limitations within this study.

### 4.1. Influence of Class Imbalance within ICF Categories on RF Performance

Even though the linking performance of our ML-based methods were consistently found to be fair to good for all the ICF categories, either specificity or sensitivity values for some of the ICF categories were very low. Those low specificity/sensitivity scores seemed to be highly influenced by the imbalance of prevalence rates of limitations and restrictions within the ICF categories. For ICF categories that had balanced rates of limitations and non-limitations, the automatic prediction method revealed satisfactory sensitivity or specificity scores. By contrast, categories with high imbalance demonstrated low F1 scores and low kappa scores, indicating that the high imbalance affects the performance of the RFs negatively, and that the prevalence rates of limitations/restrictions influences the performance strongly. The negative influence of the imbalanced data on the performance of the RFs can be explained by the low sample size. Where there are too few or too many cases of patients with limitations or restrictions in contrast to patients without limitations or restrictions, this leads to prediction methods that are biased towards the majority class [49]. Although this observation suggests that the sample size should be significantly increased, a higher sample size would still show similar imbalances due to the typical profile of LBP patients and would not solve the problem of imbalance. One of the challenges is that data imbalances may be associated with overfitting, a problem that may occur if the ML method relies too much on the “training” data [49]. This problem can be overcome by using CV techniques when tuning the RF, by using ROC-AUC as a performance metric, as it is better at handling imbalanced data than other performance metrics [49,50], and by using kappa scores as additional metric as it provides information if the performance is better than random guess by chance and therefore is also suitable metric for imbalanced data.

While Cohen’s kappa provides a valuable measure of agreement, it has its limitations, especially when dealing with imbalanced datasets or when there is a prevalence of one category over the other and should only be interpreted in combination with other performance metrics [51]. Low kappa scores for categories with high imbalance may also be explained by the so-called “Kappa paradox”, which describes cases where the individual agreement rates for certain categories might be high, but the overall agreement (observed agreement) ends up being low because of the prevalence of those categories. Therefore, the kappa scores for categories with high prevalence of one category can be biased and misleading [52,53].

For clinical use of an ML-based method, performance at high sensitivity is more desirable than high specificity. This is argued as the healthcare approach filtering out of patients with no limitations, who had been erroneously misclassified as patients with limitation, could be reached through additional testing with instruments of high specificity and lower sensitivity as long as the costs and risks of further testing are not significant [54,55]. The linking performance of the ICF categories (d530, d760, and d845) seem to fulfill these criteria as they identified the problems at high sensitivity but low specificity. Indeed, we also observed categories (d410, d415, and d430) that were identified with low sensitivity by the RF model. To improve the model’s sensitivity, we calculated a RF that considered class weights. Class weights can be used in situations where the classes are imbalanced and where the model may become biased towards the majority class and have lower sensitivity towards the minority class. By assigning higher weights to the minority class during training, the model is forced to pay more attention to these instances and can improve its ability to identify positive cases [56]. However, the detection rate of patients correctly classified with a problem could not be improved (results are not shown). Future research could consider oversampling and undersampling techniques, as these focus on balancing the data by replicating samples of the minority class or by removing samples of the majority class. Both techniques can lead to improvements in the model’s performance but can be problematic when used with CV techniques as they can lead to biased methods [49]. For this reason, these techniques were not considered for this study.

### 4.2. Performance Problem with Work-Related ICF Categories

For the two ICF categories d850 and d859, the automatic linking performance was found to be fair, despite low F1 scores and low kappa scores. Both these ICF categories measure restrictions related to work and employment, indicating that the PROMs chosen for this study did not match the criteria for an accurate linking of these work-related categories. The Tüchler study [8] demonstrated similar performance problems with work-related ICF categories. To address this problem, the Stanford Presenteeism Scale (SPS-6) [57] and the screening instrument for the identification of extensive work-related problems in patients with chronic diseases (SIMBO-C) [58] were also assessed as additional PROMs in this study. This was performed to improve linkage of the work-related ICF categories (d845, d850, and d859), but both questionnaires were not feasible in clinical practice. Many patients had major problems completing this questionnaire, which led to a high number of missing values that were not defined missing at random, and therefore were not imputable. An analysis with a subset of the data that included the work-related questionnaires SPS-6 and SIMBO-C did not reveal improvements in the performance of the RFs for either of the two ICF categories, and was therefore not considered for further analysis. This result indicates that other work-related questionnaires that have a higher correlation with the two mentioned ICF categories may be needed. In fact, improving the classification of the ML methods for these two categories is of utmost importance, as in clinical rehab practice, LBP is considered as the most common cause of work-related disability in people under 45 years of age and carries a high economic burden due to high medical expenses and workers’ compensation. [59] Better performance of these work-related categories would help medical professionals identify work-related disabilities and when work-related interventions are needed. It is worth noting that the work-related ICF categories listed in the core set for LBP may not accurately reflect work-related disabilities [60]. This could in part explain why improvement of the method could not improve the rate of the correctly classified problems for these two ICF categories.

### 4.3. Increasing Feasibility of Linking Process by Finding a Minimal Set of PROMs

The assumption that a high number of PROM variables would support the optimization of the performance of our ML methods was not confirmed by the findings of this study. One explanation for this is that too many input variables in combination with class imbalance—a typical problem for RF—could influence performance negatively by causing overfitting problems [61,62]. In addition, one must consider that collecting patient information from a high number of items may not be feasible in clinical practice [11]. In fact, when using RFE, the high number of PROM items could be considerably reduced without affecting the performance metrics for the linking process of each of the WHO-ICF activity/participation core categories for LBP. Such a reduction in the PROMs items would make the ICF linking process more feasible in a daily clinical setting. Our findings suggest that for automatic linking to the ICF core sets for LBP to be successful, a minimal set of questionnaires is necessary. Questionnaires should assess the following information: pain level (VAS), a patient’s age, items 1–4 of the PDI questionnaire, the health index of the EQ5D (EQ5D-VAS), and the AEQ. Interestingly, the items of the RMQ, which were included in the linking rules by Cieza [6] and also used for linking in our previous study [8], appeared to be of minor relevance to the RF methods used in this study. It is noteworthy that it takes patients approximately 10 min to fill out the questionnaires consistent with a set of 24 items, relative to 20 to 25 min for the full data set. Reducing the number of items further to a minimum set of 15 items would reduce the questionnaire time to approximately 5–7 min. One could argue that if using only parts of validated questionnaires for these minimal subsets, then the shortened questionnaire should be validated. However, for linking purposes, this is not necessary as the questionnaire items are only used for prediction purposes and not for building scores or other psychometric purposes. This further supports the feasibility of the linking process.

### 4.4. Performance of Novel ML Methods Compared to a Previously Published One

A direct comparison of our RF approach with a recently published linking method by Tuechler et al. [8] using the data set of either study demonstrated that RF performance could indeed be improved [8] for all categories except for categories d540 and d845. In the Tüchler data set, these two categories were found to be limited/restricted in 21 percent of the patients for category d540 and 15 percent for d845. By contrast, using the dataset from this study, we identified a limitation in d540 in 45 percent of the LBP patients and a restriction in d845 in 30 percent of the patients. This suggests that a high balance of item presentation leads to a better performance of the RFs as indicated by the higher AUC values observed. The more reliable performance of our improved methods may be explained by a higher chance of a random correct prediction. This explanation is further supported by the observations that the Tuechler et al. [8] method applied to the dataset of this study performed worse, as indicated by clearly lower AUC values for all the ICF categories investigated and also lower kappa scores for those ICF categories where the occurrence of a problem within an ICF category was found to be balanced within the sample investigated. The results from this analysis are shown in Table 3.

### 4.5. Clinical Implications

Our ML-based linking method allows one to identify problems in the ICF activity/participation core categories from a minimum item set of PROMs with a high sensitivity. If a patient is shown the results from the linking process immediately after completing the PROMs and has an opportunity to confirm or deny if the selected limitation/restriction matches with his/her impression, the information collected might be more appropriate and of higher data quality compared to a situation in which only the ICF categories are assessed (as intended with ClinFit, which was developed by the WHO Research Branch Information Tool (ClinFIT) [63]). Such a linking-related feedback approach would likely support higher data quality, despite the advantage of being more resource-efficient compared to the determination of the ICF categories by traditional external ratings. Our linking approach could further aid physicians with the assessment of functioning and disability in all health care settings, and enable more active involvement of the patients in the assessment. This approach in turn might further improve data quality and lead to higher satisfaction amongst patients. Another advantage of the linking process is the potential to use historical questionnaire-derived PROM data from previous patient visits or from other data sources, such as patient treatment efficacy studies. In such a scenario, the effectiveness of interventions for individual’s limitations or restrictions could be investigated.

### 4.6. Limitations

When splitting a dataset for ML, practical constraints, such as limited data availability, costly data acquisition, or the risk of losing important patterns or trends within the data, may make the approach unaffordable. In medical applications, where large datasets can be difficult to collect and annotate, smaller datasets are often used [64]. However, splitting smaller datasets can result in insufficient data for training and testing, leading to overfitting or the poor generalization performance of the ML method [65]. In such cases, alternative approaches, such as cross-validation or bootstrapping, may be used to evaluate the performance of the ML method [66]. In this study, a relatively small sample size was used for accurate classifications, and to avoid reducing the sample size further, we used 10-fold cross-validation and out-of-bag evaluation instead of splitting the data into a training set and test set. The small sample size also necessitated the dichotomization of the ICF categories for classification, as multinomial classification on a five-level scale would require a much larger sample size. However, the results of this study suggest that the multinomial classification of ICF categories may not be necessary in the clinical usage of the proposed linking process. Instead, rating the degree of limitation/restriction within each ICF category can be achieved if predicted limitations/restrictions are shown to the patient after successful linking. In addition, identifying whether someone is limited within an ICF category may be sufficient to trigger further assessment, e.g., team-codified assessments that would evaluate the need for rehabilitation.

The study sample consisted largely of patients with moderate pain and low disability levels, which is in accordance with a typical profile of LBP patients within secondary care settings. Because of this limitation, additional methods were developed using a sample where patients with low pain levels were excluded. An analysis of additional samples where individuals demonstrated pain levels greater than 30 and pain levels greater than 40 (levels which may be more in accordance with primary care settings), showed similar or slightly worse AUC scores for most of the ICF categories and worse kappa scores for all the ICF categories (Table 3).

This study performed the collection of PROM data and the ICF assessment guided by a psychologist on different examination days. The time between assessments likely introduced variability and consequently inflated the correlation values observed from our performance analyses. Thus, the true performance of our optimized ML algorithms may be presumably better than suggested by the findings of this study. However, as we were primarily interested in comparing the performance of different ML linking algorithms, such a procedure did not interfere with the hypothesis testing of this research. In fact, our study protocol considered that (1) a cLBP person’s problems with functioning and health are widely stable over weeks in most of the cases; (2) a study participants’ tiredness from testing after a comprehensive assessment with PROMs, body function testing, and a thorough medical examination, which could likely result in a lowered participants’ compliance with the psychologist’s ICF interview, consequently increases the likelihood of inappropriate answers; and (3) the lack of an optimum comparator ICF assessment. Even if it is conducted by well-trained and highly experienced clinical psychologists, as it was the case in our study, introducing variability seems likely. Future research will have test the true performance of our novel ML linking algorithms using an optimum comparator ICF assessment in a timely fashion. This could be best realized by confronting the person with the results from the automatically linked ICF categories immediately after completing the online PROM assessment.

## 5. Conclusions

Considering that a patient’s functioning and health perspective associated with a disease will be more important to the medical decision-making process, the automatic linking of PROMs to the categories of the comprehensive WHO-ICF core set for LBP using RFs proved to be a promising approach, if categories indicating a limitation/restriction are classified. As compared with a previously published linking method by our research group, the performance of novel RF methods proved to be superior, and is more consistent overall ICF categories. We also found that it is possible to considerably reduce the number of PROM items without decreasing the performance of the linking method, which would boost feasibility in everyday clinical practice.

Future research will have to test the true performance of our novel the ML methods utilizing an optimized “comparator” ICF assessment performed immediately after completing the PROMs in LBP persons, considering a maximum variation sampling strategy in a larger sample size. This will likely boost the performance values of our novel method and allow for accurate multinomial classifications for the impairment levels of each ICF category.

## Figures and Tables

**Table 1:** Demographic variables and distribution of the study population.

	Mean (SE) or N (%) *
n	805
Female	494 (61%)
Age (years)	48.8 (0.42)
BMI (kg/m^2^)	27.1 (0.2)
Pain (VAS)	42.61 (0.91)
AEQ PPS	3.35 (0.04)
RMQ	5.63 (0.17)
PDI	22.00 (0.56)
EQ5D score	0.76 (0.01)
EQ5D VAS	64.75 (0.72)
HADS depression	4.93 (0.15)
HADS anxiety	6.34 (0.15)
START group	low risk 114 (14%) medium risk 532 (66%) high risk 91 (11%)
Education	university degree 157 (20%) high school degree 213 (26%) professional training 325 (40%) primary school 98 (12%)
Marital status	single 117 (15%) partnership 116 (14%) married 418 (52%) divorced/widowed 135 (17%)
Employment status	employed 542 (67%) self-employed 11 (1%) retired 64 (8%) student 16 (2%) unemployed 146 (18%) other 15 (2%)

Pain (VAS) = Current pain intensity on a visual analog scale (0–100); AEQ = avoidance endurance questionnaire PPS = pain, persistence sub-scale (from 0 to 6; higher values indicate higher pain persistence during severe pain); RMQ = Roland–Morris questionnaire (from 0 to 24; higher values indicate higher disability levels); PDI = pain disability index (from 0 to 70; higher values indicate higher disability); EQ5D = European Quality of Life 5 Dimensions (score between 0 and 1; VAS between 0 and 100; higher values indicate better health); HADS = Hospital Anxiety and Depression Scale (two scores between 0 (low) and 21 (high) for either anxiety or depression), START = Subgroups for Targeted Treatment Back Screening Tool. * not specified or missing if sum of percentages =/= 100.

**Table 2:** Results of the RF methods for each ICF category. Results on the left side are based on RF methods without recursive feature elimination (RFE). Results on the right side are based on RF methods that used RFE techniques.

ICF Category	Random Forest							Random Forest with Feature Selection						
	AUC	SEN	SPE	PRE	F1	ACC	K	AUC	SEN	SPE	PRE	F1	ACC	K
d240	0.78	0.59	0.80	0.68	0.63	0.71	0.40	0.78	0.60	0.79	0.68	0.63	0.71	0.41
d410	0.76	0.30	0.93	0.67	0.41	0.75	0.28	0.77	0.31	0.93	0.66	0.41	0.75	0.29
d415	0.80	0.09	0.99	0.83	0.30	0.91	0.14	0.81	0.08	0.99	0.83	0.27	0.91	0.10
d430	0.80	0.11	0.98	0.53	0.19	0.82	0.12	0.81	0.14	0.98	0.61	0.22	0.83	0.10
d450	0.77	0.72	0.68	0.71	0.71	0.70	0.41	0.78	0.74	0.65	0.69	0.71	0.70	0.39
d530	0.76	0.97	0.15	0.82	0.89	0.81	0.17	0.78	0.97	0.20	0.83	0.90	0.82	0.18
d540	0.81	0.84	0.66	0.76	0.79	0.76	0.50	0.81	0.84	0.66	0.75	0.79	0.76	0.50
d640	0.81	0.55	0.86	0.69	0.61	0.75	0.43	0.81	0.57	0.87	0.70	0.63	0.76	0.44
d760	0.74	0.96	0.13	0.78	0.86	0.76	0.12	0.74	0.96	0.13	0.78	0.86	0.76	0.15
d845	0.75	0.85	0.41	0.73	0.78	0.69	0.28	0.75	0.86	0.39	0.73	0.78	0.69	0.25
d850	0.73	0.19	0.95	0.65	0.28	0.75	0.18	0.74	0.19	0.96	0.68	0.28	0.75	0.19
d859	0.73	0.36	0.89	0.63	0.45	0.71	0.28	0.73	0.36	0.90	0.64	0.45	0.71	0.28

AUC = Receiver operating characteristic—area under the curve; SEN = sensitivity/recall; SPE = specificity; PRE = precision; F1 = F1 score; ACC = accuracy; K = Cohen’s kappa.

**Table 3:** RF methods performance for pain cutoff subsets and for comparison with Tuechler et al. results. Note that the results for Tuechler et al., 2020 [8] are directly from the published paper, where only AUC and kappa values were presented, and ICF category d530 was excluded.

ICF	All Variables, Cutoff: Pain > 30 *				All Variables, Cutoff: Pain > 40 *				Tuechler et al., 2020 [8]: Old Algorithm			
	80 Items n = 545				80 Items n = 438				New Dataset, 32 Items n = 809		Old ** Dataset, 32 Items n = 448	
	AUC	SEN	SPE	K	AUC	SEN	SPE	K	AUC	K	AUC	K
d240	0.75	0.44	0.87	0.34	0.74	0.32	0.92	0.27	0.64	0.30	0.70	0.30
d410	0.69	0.06	0.99	0.06	0.71	0.09	0.97	0.09	0.72	0.32	0.75	0.37
d415	0.78	0.07	0.99	0.09	0.72	0.01	0.99	0.01	0.76	0.23	0.72	0.40
d430	0.77	0.01	0.99	0.01	0.79	0.01	0.99	0.01	0.73	0.30	0.73	0.29
d450	0.75	0.56	0.76	0.33	0.75	0.52	0.79	0.32	0.72	0.38	0.78	0.40
d530	0.75	0.94	0.20	0.17	0.72	0.96	0.12	0.11	-	-	-	-
d540	0.80	0.76	0.71	0.47	0.80	0.64	0.76	0.40	0.79	0.39	0.87	0.55
d640	0.82	0.41	0.93	0.39	0.80	0.30	0.94	0.28	0.75	0.36	0.71	0.34
d760	0.74	0.94	0.24	0.21	0.75	0.93	0.25	0.22	0.70	0.31	0.67	0.12
d845	0.68	0.78	0.38	0.17	0.68	0.81	0.37	0.18	0.69	0.31	0.79	0.27
d850	0.77	0.11	0.97	0.11	0.76	0.10	0.99	0.11	0.67	0.22	0.69	0.16
d859	0.70	0.17	0.95	0.15	0.71	0.16	0.96	0.16	0.65	0.31	0.61	0.20

AUC = Receiver operating characteristic—area under the curve; SEN = sensitivity/recall; SPE = specificity; K = Cohen’s kappa. * patients with pain below cut off were excluded; ** AUC values from the Tuechler et al. (2020) [8] paper, where a smaller dataset and a different algorithm was used.

**Table 4:** Confusion matrices for the RF method of each ICF category. Note that these results are based on the RF methods that did not use RFE.

ICF Category			Predicted Condition		ICF Category			Predicted Condition	
			imp.	Not imp.				imp.	Not imp.
d240	Actual condition	impaired	195	131	d410	Actual condition	impaired	70	154
		not imp.	100	361			not imp.	44	519
d415		impaired	5	74	d430		impaired	18	122
		not imp.	1	704			not imp.	17	625
d450		impaired	291	108	d530		impaired	612	20
		not imp.	126	261			not imp.	130	24
d540		impaired	368	68	d640		impaired	155	118
		not imp.	130	24			not imp.	64	442
d760		impaired	565	27	d845		impaired	390	69
		not imp.	159	28			not imp.	149	95
d850		impaired	32	131	d859		impaired	77	154
		not imp.	20	423			not imp.	45	413

**Table 5:** Performance of RF methods for different subsets of questionnaires.

ICF Category	Age; VAS; PDI 1–5; RMDQ 9,16,17,21; EQ5D 1,3; EQ5D VAS; AEQ 1–7; HADS 1,2,13 = 24 Items							Age; VAS; PDI 2–4; RMDQ 9,16,17,21; EQ5D 1,3; EQ5D VAS; HADS 1,2,13 = 15 Items						
	AUC	SEN	SPE	PRE	F1	ACC	K	AUC	SEN	SPE	PRE	F1	ACC	K
d240	0.78	0.59	0.82	0.70	0.64	0.72	0.42	0.76	0.58	0.79	0.67	0.62	0.70	0.38
d410	0.74	0.28	0.94	0.66	0.38	0.75	0.25	0.73	0.32	0.91	0.60	0.41	0.74	0.27
d415	0.77	0.05	0.99	0.75	0.30	0.90	0.08	0.73	0.05	0.99	0.60	0.30	0.90	0.08
d430	0.79	0.18	0.98	0.71	0.27	0.84	0.22	0.79	0.17	0.97	0.56	0.25	0.82	0.19
d450	0.76	0.73	0.68	0.71	0.72	0.71	0.41	0.75	0.73	0.69	0.71	0.72	0.71	0.42
d530	0.75	0.98	0.12	0.82	0.89	0.81	0.14	0.75	0.97	0.15	0.82	0.89	0.81	0.16
d540	0.78	0.85	0.60	0.73	0.78	0.74	0.46	0.78	0.86	0.58	0.72	0.78	0.73	0.45
d640	0.81	0.57	0.86	0.69	0.63	0.76	0.45	0.82	0.56	0.86	0.71	0.62	0.76	0.44
d760	0.71	0.96	0.11	0.77	0.86	0.75	0.09	0.71	0.94	0.15	0.78	0.85	0.75	0.12
d845	0.75	0.86	0.40	0.73	0.79	0.70	0.29	0.73	0.85	0.39	0.72	0.78	0.69	0.26
d850	0.74	0.17	0.96	0.69	0.25	0.75	0.17	0.74	0.20	0.97	0.77	0.31	0.76	0.22
d859	0.72	0.38	0.88	0.63	0.47	0.71	0.29	0.72	0.39	0.88	0.64	0.48	0.72	0.30

AUC = Receiver operating characteristic—area under the curve; SEN = sensitivity/recall; SPE = specificity; PRE = precision; F1 = F1 score; ACC = accuracy; K = Cohen’s kappa.

## Data Availability

The data presented in this study are available on request from the corresponding author.

## List of Abbreviations:

PROM	patient reported outcome measures
WHO	World Health Organization
ICF	International Classification of Functioning, Disability and Health
ML	machine learning
cLBP	chronic low back pain
LBP	low back pain
RF	random forest
ROC	receiver operating characteristic
AUC	area under the curve
VAS	Visual Analogue Scale
RMQ/RMDQ	Roland-Morris disability questionnaire
PDI	Pain Disability Index
EQ5D	European Quality of Life Questionnaire 5 Dimensions 5 Level Version
HADS	Hospital Anxiety and Depression Scale
AEQ	Avoidance endurance questionnaire
PPS	pain persistence behavior scale
START	Subgroups for Targeted Treatment Back Screening Tool
OOB	out of bag
CV	cross validation
RFE	recursive feature elimination
SPS-6	Stanford Presenteeism Scale
SIMBO-C	screening instrument for the identification of extensive work-related problems in patients with chronic diseases
